# Using Google Trends and Twitter for Prostate Cancer Awareness: A Comparative Analysis of Prostate Cancer Awareness Month and Breast Cancer Awareness Month

**DOI:** 10.7759/cureus.13325

**Published:** 2021-02-13

**Authors:** Bradley S Johnson, Samuel Shepard, Trevor Torgeson, Austin Johnson, Megan McMurray, Matt Vassar

**Affiliations:** 1 Office of Medical Student Research, Oklahoma State University College of Osteopathic Medicine, Tulsa, USA; 2 Department of Urology, Southern Illinois University School of Medicine, Springfield, USA; 3 Department of Psychiatry and Behavioral Sciences, Oklahoma State University Center of Health Sciences, Tulsa, USA

**Keywords:** prostate cancer awareness, google trends, breast cancer awareness

## Abstract

Background

We evaluated (1) whether the public interest in prostate cancer and prostate cancer screening increased following Prostate Cancer Awareness Month (PCAM) and (2) whether PCAM was as effective as Breast Cancer Awareness Month (BCAM) at generating public interest.

Methods

Using Google Trends, we measured search volume in PCAM and BCAM. We used the search volume in Google Trends as a proxy for changes in public interest from January 01, 2009 to December 31, 2018 worldwide, including the specific keywords: “Prostate Cancer”; “Prostate-Specific Antigen”; “Prostate Cancer Screening”; “Prostate Cancer Management”; “Breast Cancer”; “Breast Cancer Screening”; “Mammography”; and “Breast Cancer Management”. Also, we measured tweets containing “prostate cancer” and “breast cancer”. We used an autoregressive integrated moving algorithm (ARIMA) to forecast expected weekly search volumes during PCAM and BCAM. We then compared the Google Trends data from during PCAM and BCAM to the forecasted values and determined a “greater than expected” range.

Results

The mean pooled percent increase in tweets associated with “prostate cancer” during PCAM from 2012 through 2018 was 15.9% (95% CI, -1% - 33%). The mean pooled percent increase in tweets associated with “breast cancer” during BCAM from 2012 through 2018 was 318.5% (95% CI, 268% - 369%). BCAM was associated with a 302.6% greater effect on increasing tweets referencing the disease of interest than PCAM from 2012-2018. “Breast cancer” Google searches were found to be 36.7% (95% CI, 34% - 39%) more frequent than “prostate cancer” per month from 2009-2019. Google Searches for “breast cancer screening” were 29.6% (95% CI, 28% - 31%) greater than “prostate cancer screening”.

Conclusions

Our results indicate that PCAM is not generating substantial internet interest, especially when compared to BCAM. The search volume for Google Trends search terms related to PCAM was less than BCAM in every comparison, and Twitter indicated only a slight increase of Tweets during the month of PCAM. Suggestions are provided to improve the effect of PCAM and men’s health.

## Introduction

In 2021, prostate cancer is expected to affect nearly 250,000 men in the United States of which one in seven men with prostate cancer will die from this disease [1]. Fortunately, early detection correlates with nearly 100% of men with prostate cancer reaching the five-year relative survival rate [2]. For prostate cancer to be detected early, men must be actively screened. Men who are properly informed about prostate cancer screening are most likely to receive screening; thus, awareness can be life-saving [3].

With the current rise of social media, prostate cancer awareness messages are no longer limited to traditional media outlets. Media coverage of prostate cancer, including prostate cancer awareness month (PCAM) and its associated events, attempts to promote the benefits of screening, to educate men about disease prevention, and to campaign for prostate cancer research funding [4, 5]. PCAM specifically may play a role in the current trend of improved patient outcomes as prostate cancer mortality rates have decreased two-fold since the start of PCAM [5, 6]. Even with the relative success of PCAM in promoting awareness, PCAM has room to improve. For example, prostate cancer and breast cancer are the most commonly studied types of men’s and women’s cancers on social media; however, trends indicate that breast cancer awareness month (BCAM) has performed substantially better at spreading awareness via the internet than PCAM (2004-2009) [7, 8]. A recent *World Journal of Urology* study found that #breastcancer was tweeted 231,258 times and #prostatecancer only 44,584 times between January 2015 to July 2015 [9].

Although prostate cancer and breast cancer share common features -- such as being the most common hormonal-dependent cancers with similar prognosis and survival rates -- they do not necessarily generate similar attention or awareness efforts [1,10-12]. By using Google Trends and Twitter, we assessed the utility of PCAM in promoting awareness and its relationship to prostate cancer screening and prevention. To provide a comparison, we analyzed the relative search interests of a well-established cancer awareness month -- BCAM -- in October.

## Materials and methods

Using Google Trends (https://google.com/trends, Google LLC, Mountainview, CA, USA), we measured the relative public interest in PCAM and BCAM. We also used Sprout Social (https://sproutsocial.com/, Sprout Social, Chicago, USA) to measure social media activity by searching for tweets containing “prostate cancer” and “breast cancer” on Twitter (www. twitter.com, Twitter, Inc., San Francisco, CA, USA).

Twitter

The number of tweets containing each term was then extracted using Sprout Social and the mean number of tweets per day, per month was calculated. We then compared the mean number of tweets per day, per month for the specific awareness month (September for prostate cancer and October for breast cancer) with the mean number of tweets per day, per month from the other 11 months. This process was completed for both “prostate cancer” and “breast cancer”. All calculations were made using Stata 15.1 (StataCorp LLC, College Station, TX, USA).

Google Trends

We used the search volume in Google Trends as a proxy for changes in public interest from January 01, 2009, to December 31, 2018, worldwide, including the specific keywords: “Prostate Cancer”, “Prostate-Specific Antigen”, “Prostate Cancer Screening”, “Prostate Cancer Management”, “Breast Cancer”, “Breast Cancer Screening”, “Mammography”, and “Breast Cancer Management”. These keywords were selected because out of the keywords related to awareness and screening, they had the greatest number of search inquires. Google Trends measures relative search interest by normalizing search volumes to range from 0-100 with 100 being the highest search volume within the applied filters. Additionally, we compared relative search volumes between BCAM and PCAM through keywords that corresponded between the two months. These comparisons included "breast cancer" and "prostate cancer", "prostate cancer screening" and "breast cancer screening", "prostate cancer management" and "breast cancer management", and "mammography" and "prostate-specific antigen". Each group was compared within the same Google Trends analysis.

Secondarily for 2019, we used an autoregressive integrated moving algorithm (ARIMA) to forecast expected weekly search volumes during PCAM and BCAM. We used weekly search data from January 1, 2019 to August 31, 2019 for PCAM and January 1, 2019 to September 30, 2019 for BCAM for the ARIMA model. We then compared the Google Trends data from during PCAM and BCAM to the forecasted values and determined a “greater than expected” range. This value was used to show whether search volumes related to PCAM or BCAM were associated with an increase in public interest. R version 3.2.1 (R Foundation, Vienna, Austria) was used to run the ARIMA model and Stata 15.1 was used for all other analyses. 

## Results

Twitter results

The mean pooled percent increase in tweets associated with “prostate cancer” during PCAM from 2012 through 2018 was 15.9% (95% CI, -1% - 33%). The mean pooled percent increase in tweets associated with “breast cancer” during BCAM from 2012 through 2018 was 318.5% (95% CI, 268% - 369%). BCAM was associated with a 302.6% greater effect on increasing tweets referencing the disease of interest than PCAM from 2012-2018. A complete depiction of Twitter results can be found in Table 1.

**Table 1:** Percent increase of tweets for "prostate cancer" and " breast cancer" during their respective awareness month PCAM: Prostate Cancer Awareness Month; BCAM: Breast Cancer Awareness Month

Year	PCAM (September)	95% CI	BCAM (October)	95% CI
2012	16.4%	-7% - 31%	338.1%	150% - 1687%
2013	21.3%	-3% - 61%	352.8%	153% - 2066%
2014	27.0%	3% - 66%	355.0%	154% - 2111%
2015	40.0%	14% - 81%	382.0%	160% - 3164%
2016	10.5%	-11% - 46%	311.0%	143% - 1242%
2017	2.3%	-19% - 39%	233.1%	117% - 618%
2018	26.9%	4% - 63%	257.3%	126% - 748%
The number of tweets referencing "prostate cancer" during PCAM and "breast cancer" during BCAM were compared to the mean of the other 11 months for each year.				

Google Trends results

**Figure 1:**
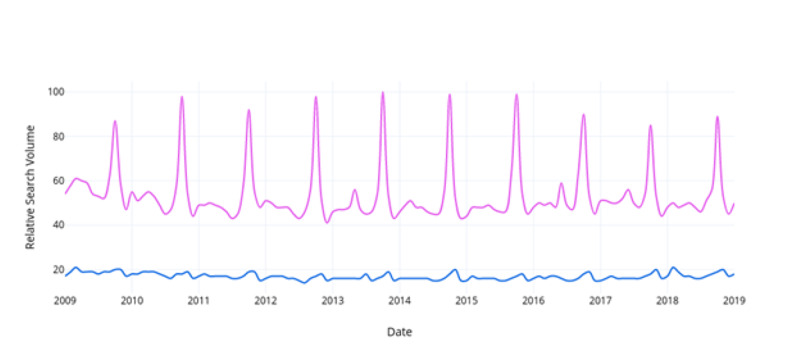
Comparison of the relative search interest for prostate cancer vs. breast cancer Breast cancer is signified in pink and prostate cancer is signified in blue.

**Figure 2:**
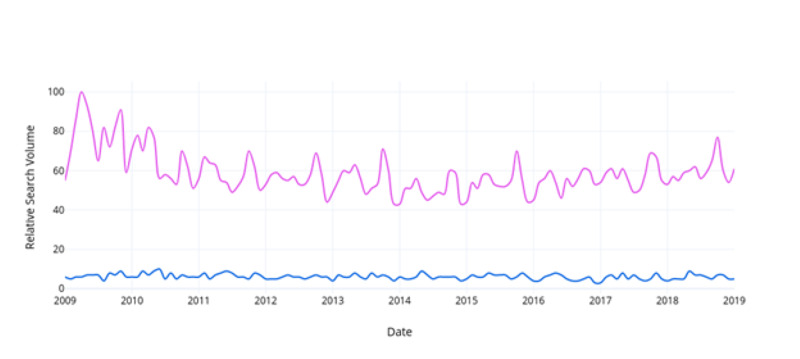
Comparison of the relative search interest for prostate cancer management vs. breast cancer management Breast cancer management is signified in pink and prostate cancer management is signified in blue.

**Figure 3:**
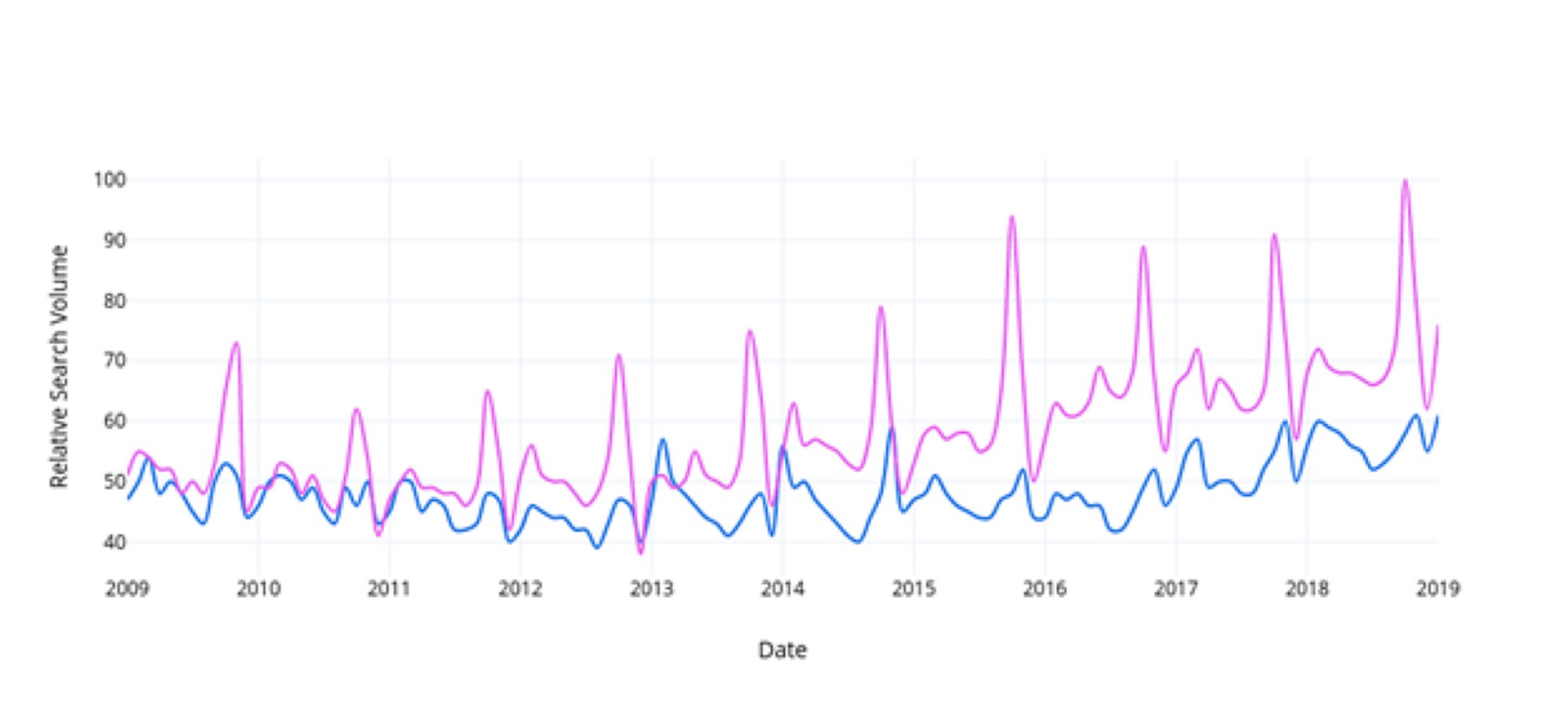
Comparison of the relative search interest for prostate-specific antigen vs. mammography Mammography is signified in pink and prostate-specific antigen is signified in blue.

**Figure 4:**
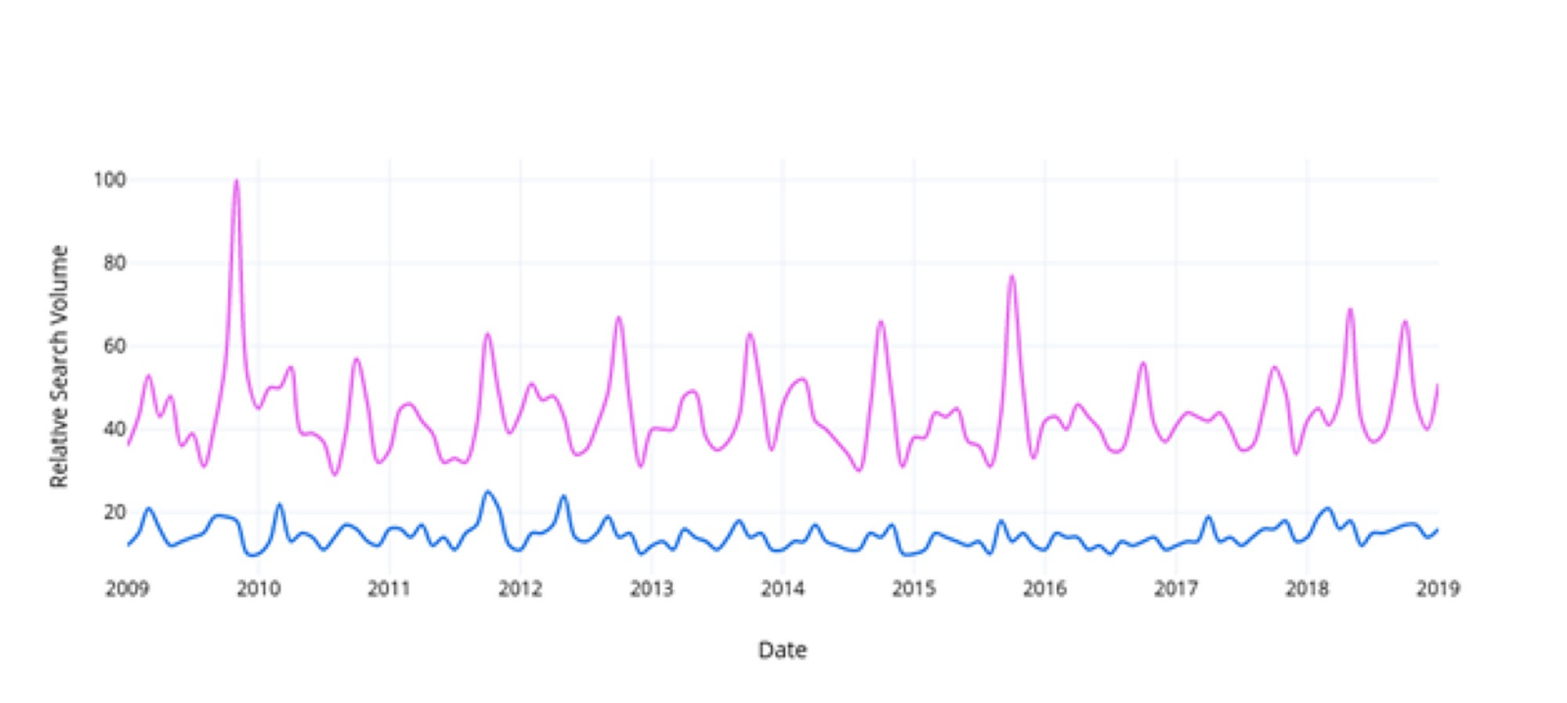
Comparison of the relative search interest for prostate cancer screening vs. breast cancer screening Breast cancer screening is signified in pink and prostate cancer screening is signified in blue.

When comparing relative search volumes for prostate cancer and breast cancer within the same Google Trends search, “breast cancer” searches were found to be 36.7% (95% CI, 34% - 39%) more frequent than “prostate cancer” per month from 2009-2019 (Figure 1). Searches for “breast cancer management” were 52.8% (95% CI, 51% - 55%) greater than “prostate cancer management” (Figure 2). Searches for “mammography” were 10.2% (95% CI, 9% - 12%) greater than “prostate-specific antigen” (Figure 3). Searches for “breast cancer screening” were 29.6% (95% CI, 28% - 31%) greater than “prostate cancer screening” (Figure 4). In 2019, the above search terms were compared to an expected search interest calculated from the ARIMA model. Overall, BCAM was shown to have a greater effect on search interest than PCAM (Table 2). 

**Table 2:** Weekly Ranges for Greater Than Expected Percentages During PCAM and BCAM in 2019 PCAM: Prostate Cancer Awareness Month; BCAM: Breast Cancer Awareness Month

Search Term	PCAM (September)	BCAM (October)
"Prostate Cancer" / "Breast Cancer"	-2.2% - 10.8%	13.8% - 46.3%
"Prostate / Breast" Cancer Management	1.0% - 46.4%	13.1% - 25.6%
"Prostate / Breast" Cancer Screening	4.6% - 23.6%	16.2% - 35.2%
"Prostate-Specific Antigen" / Mammography	10.6% - 18.6%	19.8% - 30.8%
Weekly greater than expected search ranges were calculated using an autoregressive integrated moving algorithm (ARIMA). Observed search interest from each week during the respective awareness month was compared to the expected search interest calculated from the ARIMA model.		

## Discussion

Our results demonstrate that PCAM’s ability to promote awareness regarding prostate cancer is minimal, especially when compared to BCAM. BCAM’s overall Google search volume and Twitter interest were both substantially larger than PCAM, as Twitter indicated only a slight increase of tweets during the month of PCAM, and Google Trends search terms related to PCAM were less than BCAM in every comparison. Lastly, our ARIMA forecast analysis demonstrated increases in Google Trends search volumes above expected for PCAM and BCAM in 2019, but BCAM’s search volume increases were greater than PCAM’s. 

Our findings -- which used Google Trends and Twitter over a 10 year period of time -- expand upon previous findings that deployed the Yahoo! search engine to measure search interest during PCAM from 2001 to 2003 [13]. The low level of online interest in PCAM is disappointing given the potential effects that successful awareness months can have on the promotion of health awareness and screening. For example, the percentage of American women who received mammograms doubled 10 years after the start of BCAM [14]. Research suggests that the difference in media interest between breast cancer and prostate cancer may contribute to differences in screening prevalence. In 2015, 50% of American women older than 40 received a mammogram in the past year, whereas 32% of American men older than 40 were tested for their Prostate-Specific Antigen (PSA) [15, 16]. Although increased media interest may be correlated to increased screening prevalence, the controversy of prostate cancer screening through PSA testing may also be involved. In 2012, United States Preventive Services Task Force (USPSTF) recommended against PSA testing for prostate cancer and downgraded PSA testing to a "D" grade [17]. This change may be causing many primary care physicians and urologists to not promote prostate cancer screening. However, some experts believe that this update has lowered the number of prostate cancer patients being treated early, resulting in an increase in metastatic prostate cancer cases [18]. Prostate cancer screening may be debated, but an effort to promote prostate cancer awareness is still a worthy endeavor and crucial to the advancement of prostate cancer treatments. Overall, multiple hypotheses exist regarding why PCAM has received less media interest than BCAM, including disparities in funding and expenditures, gender differences in perceptions of health, and the lack of coordination between prostate cancer awareness initiatives. Regardless of the cause, because prostate and breast cancer have similar prevalence and mortality rates [19], an investigation for solutions to improve the effectiveness of PCAM is necessary. 

Additionally, because evidence suggests that many men are not comfortable discussing health issues, these men may be less likely to advocate for men’s health online or at other events during PCAM. A survey by Cleveland Clinic’s health campaign “MENtion It” found that only 10% of men were willing to discuss their cancer diagnosis with a friend, and only 54% felt comfortable talking about their genitourinary problems with a physician [20]. A 2019 survey found that, of 2,000 men, those who value appearing “tough” regarding their health were more likely to have a higher frequency of health risks [21]. There is hope for change: a 2018 study reported men would respond to a gender-sensitized, health promotion program devoted to them [22]. PCAM should focus on ending the stigma that men who care about, and who promote health, are weak. Research shows that men are willing to respond to masculine motivation to change poor health behaviors, such as using nostalgia from previous sports experiences [22]. Perhaps, PCAM can use these masculine-focused strategies to change the negative stigma of men’s health advocacy. 

In addition, inconsistencies exist in prostate cancer and men’s health awareness months worldwide. For the UK, Australia, parts of the United States, and others, “Movember” is an awareness campaign where men grow out their facial hair for men’s health, including prostate cancer, during the month of November [23, 24]. Different resources for spreading prostate cancer awareness distributed to PCAM and “Movember” may have a reduced effect when compared to having only one initiative in which resources could be invested. Additionally, the lack of standardization of PCAM internationally may limit opportunities for collaboration between the entities sponsoring these awareness months. Similar standardization issues are not a problem for BCAM, which has been consistent internationally as an October event. 

Although Google Trends and Twitter are commonly accepted and widely used in the scientific community, the utility of these tools should be viewed in light of their limitations. Given the ‘relative search volume’ algorithm used by Google Trends, it may be misleading to compare levels of interest on two different topics. Although our selected keywords were among the most popular search inquiries, we recognize that there may be other keywords capable of assessing public interest that were not included in our sample. We are also aware that Google -- although it is the most popular search engine -- may not display a complete representation of public interest in a specific topic. The public uses many other tools to gather and disseminate important health-related information, thus our results may be subject to sampling bias. 

## Conclusions

In conclusion, results from our study indicate that PCAM has not generated substantial public interest in prostate cancer when compared to BCAM’s ability to generate interest in breast cancer. To improve these outcomes, we outline a set of suggestions that take into account more coordinated international efforts and focus on the destigmatization of men’s health.
